# Strategies to reduce genetic mosaicism following CRISPR-mediated genome edition in bovine embryos

**DOI:** 10.1038/s41598-019-51366-8

**Published:** 2019-10-17

**Authors:** I. Lamas-Toranzo, B. Galiano-Cogolludo, F. Cornudella-Ardiaca, J. Cobos-Figueroa, O. Ousinde, P. Bermejo-Álvarez

**Affiliations:** Animal Reproduction Department, INIA, Madrid, Spain

**Keywords:** Genetic engineering, Embryology

## Abstract

Genetic mosaicism is the presence of more than two alleles on an individual and it is commonly observed following CRISPR microinjection of zygotes. This phenomenon appears when DNA replication precedes CRISPR-mediated genome edition and it is undesirable because it reduces greatly the odds for direct KO generation by randomly generated indels. In this study, we have developed alternative protocols to reduce mosaicism rates following CRISPR-mediated genome edition in bovine. In a preliminary study we observed by EdU incorporation that DNA replication has already occurred at the conventional microinjection time (20 hpi). Aiming to reduce mosaicism appearance, we have developed three alternative microinjection protocols: early zygote microinjection (10 hpi RNA) or oocyte microinjection before fertilization with either RNA or Ribonucleoprotein delivery (0 hpi RNA or 0 hpi RNP). All three alternative microinjection protocols resulted in similar blastocyst and genome edition rates compared to the conventional 20 hpi group, whereas mosaicism rates were significantly reduced in all early delivery groups (~10–30% of edited embryos being mosaic depending on the loci) compared to conventional 20 hpi microinjection (100% mosaicism rate). These strategies constitute an efficient way to reduce the number of indels, increasing the odds for direct KO generation.

## Introduction

Genome modification at specific loci allows the ablation (knock-out, KO) or insertion (knock-in, KI) of specific DNA sequences to unequivocally assess the role of a specific gene on a particular physiological process or to alter the phenotype of an animal for diverse purposes. Unfortunately, technical limitations have largely restricted targeted genome modification in mammals to the mouse model. These limitations derived from the extremely low efficiency of the only available technique for targeted mutagenesis, homologous recombination (HR)^1^, which impedes its direct application on embryos. The low efficiencies of HR could only be bypassed by the use of embryonic stem cells (ESC) as an intermediary to generate targeted genetically modified mice^2^. Later, the development of somatic cell nuclear transfer (SCNT) allowed the use of somatic cells as intermediaries for HR to generate genetically modified animals in other species where truly pluripotent ESC were not available^3^, but the complexity and inefficiency of SCNT has impeded its widespread use. The advent of site-specific endonucleases has situated genome modification at an attainable distance in these species, empowering scientist to perform loss-of-function experiments or to generate genetically modified animals for different applications^4^. Among the different site-specific endonucleases, CRISPR, the last technology to be developed has become the method of choice given its ease of use and flexibility^5^.

CRISPR technology allows KO generation in a single-step by microinjection at the zygote stage^6^. For this purpose, CRISPR is directed to the beginning of the coding region of the target gene, where it will induce a double-strand break (DSB). The DSB can be repaired by either HR, which reconstitutes the target site allowing CRISPR recognition and thereby the generation of another DSB, or by non-homologous end joining repair (NHEJ), which often generates random insertions or deletions (indels) at the target site. Those indels constitute a stable mutation, as they impede target recognition by CRISPR, and can produce KO alleles, as those indels not multiple of three disrupt the open reading frame (ORF) of the target gene, leading to a truncated protein. However, KO generation in one step (i.e. one pregnancy) requires all alleles harboured by a given individual to be KO (i.e., not multiple of three) and, therefore, a reduction in the number of alleles generated greatly increases the odds for direct KO generation.

The possible generation of more than two alleles per individual following CRISPR edition was initially overlooked, as seminal work in mice did not report this phenomenon^6^, and because in this species breeding of founders to obtain an heterozygous F1 generation is the routine protocol for experimental purposes. Under ideal conditions, zygote genome edition should occur at the 2n2c stage, resulting in two indels (alleles). However, DNA replication occurs soon after fertilization, before pronuclei fusion, transitioning to the 2n4c stage where genome edition can result into more than two alleles, a phenomenon called genetic mosaicism. DNA edition may also occur at later developmental stages (after cleavage) if both unedited alleles and active ribonucleoprotein are present. Mosaic individuals are composed by more than one genetic cell type and have been consistently found in most publications that have performed allele screening following CRISPR direct injection in zygotes of diverse species such as mice^7,8^, pigs^9–19^, goats^20^, sheep^21–23^, cattle^25^ and rabbits^26–34^. In this article, we report strategies to reduce genetic mosaicism following CRISPR edition of bovine embryos based on early delivery of CRISPR components.

## Results

### Development of a shortened IVF protocol

**Figure 1:**
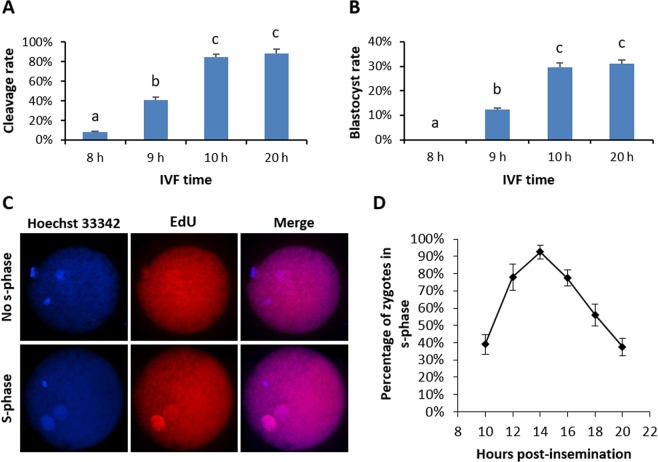
Developmental rates following shortened gamete co-incubation times and kinetics of S-phase in bovine IVF zygotes. (**A**) Cleavage and **(B**) blastocyst rates obtained following gamete co-incubation for 8, 9, 10 or 20 h. (**C**) Representative pictures of S-phase analysis in bovine zygotes following EdU protocol; upper images show a zygote that is not replicating its DNA (EdU negative), lower images depict a zygote on S-phase (EdU positive). (**D**) Percentages of zygotes replicating its DNA from 10 to 20 hpi in 2 h intervals.

Conventional protocols of bovine *in vitro* fertilization (IVF) entail the co-incubation of cumulus-oocyte complexes and spermatozoa for ~20 h^35^. Following co-incubation, cumulus cells can be removed and then, the ooplasm of the presumptive zygotes can be clearly visualized and microinjected. These IVF and micromanipulation conditions have been optimized to attain high developmental rates, but the delivery of CRISPR components by microinjection at 20 h after the onset of IVF resulted in all edited embryos being mosaic (Table 1). Aiming to explore the different possibilities of an earlier delivery of CRISPR components, we first established the minimum gamete co-incubation time to achieve normal developmental rates in a preliminary experiment. For this aim we reduced IVF time to 8, 9 or 10 h, observing that, in the case of the semen used in these experiments, 10 h was the minimum gamete co-incubation time to achieve similar developmental rates to the conventional 20 h IVF protocol (Fig. 1A,B and Table S1).

**Table 1:** Genome edition and mosaicism rates following the alternative protocols tested. Different superscript letters indicate significant differences based on Chi-square test (p < 0.05).

Group	No. of embryos genotyped by PCR sequencing	No. of embryos edited (%)	No. of embryos genotyped by clonal sequencing	No. of mosaic embryos (%)
RNP-injected 0 hpi	23	20 (87.0)	20	6 (30.0)^a^
mRNA-injected 0 hpi	25	22 (88.0)	20	6 (30.0)^a^
mRNA-injected 10 hpi	24	20 (83.3)	20	7 (35.0)^a^
RNP-injected 20 hpi	6	5 (83.3)	5	5 (100)^b^
mRNA-injected 20 hpi	25	21 (84.0)	10	10 (100)^b^

### Characterization of S-phase in bovine zygotes

Once the minimum co-incubation time was established, we analysed the timing of DNA replication in bovine IVF derived zygotes from the minimum IVF time (10 hours post-insemination –hpi-) to 20 hpi in 2 h intervals by 5-Ethynyl-2′-deoxyuridine (EdU) incorporation (Fig. 1C). The data obtained show that DNA replication occurs short after fertilization, with ~40% of the zygotes already replicating its DNA at 10 hpi (Fig. 1D). Four hours later (14 hpi) most zygotes were on S-phase and at the time point when gamete co-incubation ceases in conventional IVF procotols (20 hpi), ~40% of the zygotes are on S-phase. These kinetics suggest that S-phase spans for about 10 h and that by the end of the conventional gamete co-incubation (20 hpi), most zygotes have replicated its DNA or are very close to end their S-phase. In this context, an earlier CRISPR deliver is required to prevent the appearance of more than two alleles in a given embryo.

### Developmental outcomes following different microinjection times

**Figure 2:**
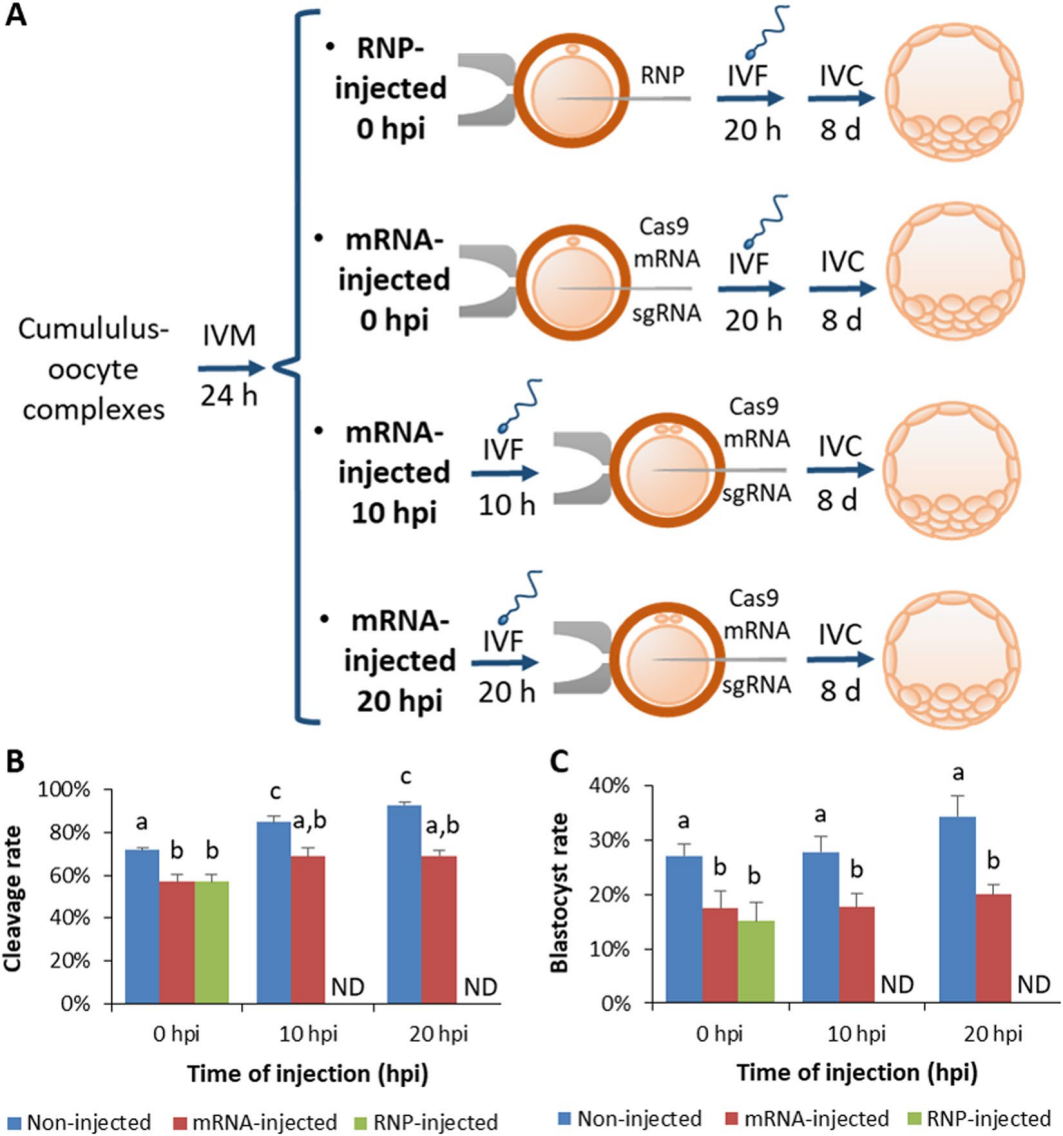
Developmental rates following the alternative microinjection protocols tested. **(A**) Schematic representation of the three alternative protocols tested and the conventional 20 hpi protocol. (**B**) Cleavage and (**C**) blastocyst rates obtained following the alternative protocols tested with or without microinjection. Different letters indicate significant differences based on ANOVA (p < 0.05).

Two early microinjection strategies were tested: (1) microinjection right after a shortened IVF protocol (10 hpi) and (2) oocyte microinjection (0 hpi) followed by IVF of cumulus-free oocytes (Fig. 2A). Embryo developmental rates following these alternative protocols were initially assessed without performing microinjection. As previously tested in a smaller experiment (Fig. 1A,B), 10 hpi protocol yielded similar cleavage and blastocyst rate than the 20 hpi control (Fig. 2B,C and Table S2). When oocytes were fertilized without cumulus cells (0 hpi group), cleavage rate was reduced compared to both groups fertilized with cumulus cells (Fig. 2B, ANOVA p < 0.05), but no significant differences were found on blastocyst yield between the three non-microinjected groups (Fig. 2C and Table S2). In order to determine the developmental effects of the microinjection procedure and the possible toxicity of CRISPR components without the interference of a possible detrimental effect of the genome edition *per se*, a genomic target locus located in a non-coding region was chosen. The microinjection of CRISPR components (mRNA and sgRNA at 0, 10 or 20 hpi and ribonucleoprotein at 0 hpi) in bovine oocytes or zygotes caused a statistically significant reduction in cleavage and blastocyst rates in all four groups analysed compared to their corresponding (time-matched) non-microinjected control (Fig. 2B,C, ANOVA p < 0.05). No significant differences were observed in blastocyst rates between all microinjected groups (Fig. 2C and Table S2).

### Genome edition and mosaicism rates following different microinjection times

**Figure 3:**
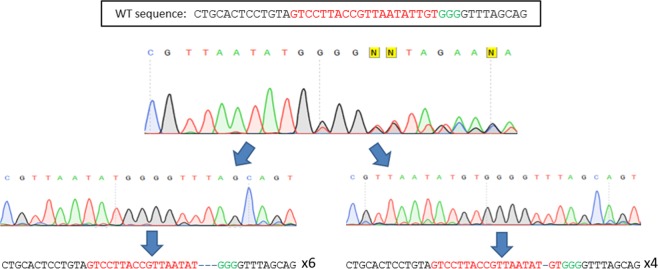
PCR and clonal sequencing of an edited non-mosaic embryo. Upper image show the sequencing reaction of a PCR product, mixed peaks indicates edition but do not allow allele discrimination. Lower images show the sequencing reaction of individual alleles following clonal sequencing.

Genome edition rates were assessed in all four microinjected groups (3 microinjected with mRNA + sgRNA at 0, 10 and 20 hpi, and 1 microinjected with ribonucleoprotein at 0 hpi) and in 5 embryos obtained from a replicate microinjected with ribonucleoprotein at 20 hpi (Table 1). For this aim, a PCR product including CRISPR target site was amplified and sequenced in at least 20 blastocysts per group. All groups displayed similar genome edition rates, with more than 80% of the blastocyst produced from microinjected oocytes or zygotes being edited at the target site. This sequencing strategy allowed to distinguish between edited and not-edited embryos, but does not provide the number of alleles harboured by a given embryo, as mixed sequencing peaks preclude allele identification (Fig. 3). Allele identification was achieved by clonal sequencing, analysing 10 colonies per embryo in 20 embryos on each of the three early microinjection groups (600 sequences) and 10 and 5 in 20 hpi group microinjected with mRNA or ribonucleoprotein, respectively (150 sequences) (Fig. S1). Mosaicism rates were significantly reduced in all early delivery groups compared to 20 hpi (70% reduction from 100% to ~30%, Table 1), but no differences were noted between 0 or 10 hpi or between CRISPR injection formats. The number of embryos carrying WT (not edited) alleles and carrying only alleles formed by indels not multiple of three (there would be KO embryos if the target was allocated on a coding region) are shown in Table 2. As expected the percentage of embryos carrying WT alleles was significantly higher in the group where all embryos were mosaic (20 hpi).

**Table 2:** Percentage of embryos containing unedited WT alleles or only indels non-multiple of 3. Different superscript letters indicate significant differences based on Chi-square test (p < 0.05) between the four microinjection groups.

Group	No. of edited embryos	No. of edited non-mosaic embryos (%)	No. of embryos containing an unedited WT allele (%)	No. of embryos containing only indels non-multiple of 3 (%)
RNP-injected 0 hpi	20	14 (70.0)^a^	8 (40.0)^a,b^	3 (15.0)
mRNA-injected 0 hpi	20	14 (70.0)^a^	6 (30.0)^a^	6 (30.0)
mRNA-injected 10 hpi	20	13 (65.0)^a^	6 (30)^a^	6 (30.0)
RNP-injected 20 hpi	5	0 (0)^n.d.^	4 (80.0)^n.d.^	0 (0)
mRNA-injected 20 hpi	10	0 (0)^b^	8 (80.0)^b^	1 (10.0)

### Genome edition and mosaicism rates targeting multiple coding loci

To further test the most user-friendly of the two alternative microinjection protocols developed (0 hpi), we analysed genome edition and mosaicism rates in blastocysts derived from mRNA (n = 26) or ribonucleoprotein microinjections (n = 23) targeting two coding loci simultaneously. These loci were localized at the beginning of the coding region of two genes encoding for the proteins *CSN2* and *PAEP*, both present in cow milk and responsible for allergic reactions in humans. In this experiment genome edition rates were around or above 90%, and all embryos edited on one locus were also edited in the other except for one in the RNP group (Table 3). Mosaicism rates were reduced to 12–24%, and again no statistically significant differences were found on edition, mosaicism or KO generation rates between the groups injected with mRNA or ribonucleoprotein (Tables 3 and 4). Embryos were deemed as KO for a gene when all alleles detected were formed by indels not multiple of three.

**Table 3:** Genome edition and mosaicism rates following dual targeting (*PAEP* and *CSN2*) by mRNA or RNP microinjections at 0 hpi. No significant differences were observed based on Chi-square test (p > 0.05).

Group	No. of embryos genotyped by PCR sequencing for PAEP	No. of embryos edited for PAEP (%)	No. of mosaic embryos for PAEP (%)	No. of embryos genotyped by PCR sequencing for CSN2	No. of embryos edited for CSN2 (%)	No. of mosaic embryos for CSN2 (%)
RNP-injected 0 hpi	23	21 (91.3)	3 (14.3)	23	20 (87.0)	3 (15.0)
mRNA-injected 0 hpi	26	25 (96.2)	3 (12.0)	26	25 (96.2)	6 (24.0)

**Table 4:** Percentage of KO embryos (i.e., harbouring only frame-disrupting alleles) following dual targeting (*PAEP* and *CSN2*) by mRNA or RNP microinjections at 0 hpi.

Group	No. of embryos edited for PAEP	No. of KO embryos for PAEP (%)	No. of embryos edited for CSN2	No. of KO embryos for CSN2 (%)	No. of embryos edited for both PAEP and CSN2	No. of KO embryos for both PAEP and CSN2 (%)
RNP-injected 0 hpi	21	11 (52.3)	20	8 (40.0)	20	5 (25.0)
mRNA-injected 0 hpi	25	8 (32.0)	25	14 (56.0)	25	5 (20.0)

## Discussion

Mosaicism constitutes a major problem for direct genome modification following the microinjection of CRISPR microinjection in zygotes. The presence of more than two alleles in a given individual reduces greatly the chances of KO generation in one step, without the need of subsequent breeding of founder individuals, as all alleles generated must disrupt the ORF of the target gene. The generation of a KO individual in a single pregnancy is especially relevant in livestock species, where, in contrast to mice, generation intervals can be counted by years. In this perspective, the requirement of breeding mosaic founders for the generation of bi-allelic animals impede the culmination of genome modification projects within a reasonable time frame in these species. Besides, KO generation in one step would allow to elucidate gene function during embryo development without the need of a colony of heterozygous founders. A myriad of publications that have performed allele screening following CRISPR direct injection in zygotes of diverse species such as mice^7,8^, pigs^9–19^, goats^20^, sheep^21–23^, cattle^25^ and rabbits^26–34^ have encountered this problem and yet, probably due to the time and resource consuming allele screening, no study has systemically analysed mosaicism rates following different protocols.

The analysis of S-phase on bovine zygotes show that DNA replicates short after fertilization, providing a physiological explanation for the high mosaicism rates obtained following conventional microinjection times, as four DNA copies of the target sequence will be present when genome edition occurs. The timing and duration of S-phase in bovine *in vitro* produced zygotes overtly agrees with previous studies^36,37^, and although there are differences between bulls in both the onset of S-phase^37^ and cleavage division^38^, the protocols tested in this study could be applied to the use of different sires with minor (10 hpi) or no (0 hpi) modifications. Likewise, early delivery of CRISPR components is expected to reduce mosaicism rates in other species. In this context, earlier CRISPR delivery has been observed to diminish mosaicism rates following CRISPR electroporation in mice^39^, and small data sets from sheep^24^ and human^40^ studies also suggest a reduction.

The early microinjection protocols tested achieved a ~70–90% reduction in mosaicism rates. This reduction constitutes a remarkable advance for direct KO generation, but mosaicism was not completely abolished. The use of ribonucleoprotein instead of RNAs did not reduced further mosaicism rates, suggesting that the time required for RNA translation and ribonucleoprotein assembly was not a crucial factor for mosaicism appearance. Somehow surprisingly, CRISPR delivery to bovine oocytes (i.e., before fertilization) did not decrease mosaicism rates further compared to the delivery to presumptive zygotes right after a reduced IVF protocol (10 hpi), suggesting that CRISPR activity is very low before pronuclei formation. A possible explanation for this result is that CRISPR may not be able to recognize its target locus before some degree of chromatin decondensation has been achieved in the pronuclei, as chromatin accessibility exerts a great impact on Cas9 binding *in vivo*^41,42^. In this sense, the high degree of DNA condensation in matured oocytes and especially on spermatozoa^43^ may impede an earlier CRISPR-mediated genome edition.

In conclusion, early delivery of CRISPR components to bovine oocytes prior to IVF or zygotes following a shortened IVF reduces mosaicism rates from 100% to ~10–30% while achieving similar genome edition and developmental rates. Oocyte microinjection is more convenient schedule-wise than microinjection following a 10 h IVF protocol and both RNA or ribonucleoprotein delivery formats can be used, as both achieved similar results.

## Methods

### Bovine *in vitro* production

Ovaries were collected at local slaughterhouse and transported to the laboratory within 2 h. Cumulus-oocyte complexes (COCs) were collected from 2 to 8 mm diameter follicles and selected based on conventional morphological criteria^44^. *In vitro* maturation (IVM) was performed in TCM-199 supplemented with 10% (v/v) fetal calf serum (FCS) and 10 ng/ml epidermal growth factor at 39 °C under an atmosphere of 5% CO_2_ in air with maximum humidity for 24 h. IVF was performed with frozen-thawed spermatozoa from a single sire selected by Bovipure® (Nidacon). Spermatozoa were diluted to a final concentration of 10^6^ spermatozoa/ml and were co-incubated with mature oocytes in TALP medium at 39 °C under an atmosphere of 5% CO_2_ in air with maximum humidity. Different co-incubation times were initially tested (8, 9, 10 and 20 h) to determine the minimum co-incubation time (>80 embryos/group in two independent replicates). For microinjection experiments, gametes were co-incubated for 10 (10 hpi group) or 20 h (0 and 20 hpi groups) (Fig. 2A). Microinjected and non-microinjected oocytes of 0 hpi groups were denuded by vortexing in PBS supplemented with 300 µg/ml hyaluronidase for 3 min prior to fertilization, in order to visualize the ooplasm membrane for microinjection. In 10 and 20 hpi groups intact COCs were used for IVF and cumulus cells were removed at the end of the co-incubation time by vortexing in PBS for 3 min. Cumulus-free presumptive zygotes were cultured *in vitro* in SOF media supplemented with 5% FCS under an atmosphere of 5% CO_2_ and 5% O_2_ in air with maximum humidity. Cleavage rates were assessed at 48 hpi and blastocyst yield was recorded 9 days post-insemination (dpi) in 3–4 independent replicates per group. Statistical differences in developmental rates were assessed by One Way ANOVA using SigmaStat software.

### Assessment of DNA replication in bovine zygotes

DNA synthesis was detected by the incorporation of 5-Ethynyl-2′-deoxyuridine (EdU) (Click-iT EdU HCS assay kit, Invitrogen). Briefly, following 10, 12, 14, 18 and 20 hpi, 243 presumptive zygotes from 3 independent replicates were vortexed 3 min to remove cumulus cells and incubated in 50 µM EdU for 30 min^45^. EdU labelling was detected following manufacturer instructions and pronuclei were counterstained with 10 µg/ml Hoechst. Zygotes were observed on an epifluorescence inverted microscope (Nikon Eclipse TE300) and considered to be on S-phase stage when they displayed at least one pronucleus labelled with EdU.

### CRISPR/Cas9 microinjection

Details for sgRNAs are provided on Table S3. sgRNAs were synthesized and purified using Guide-it sgRNA *In Vitro* Transcription Kit® (Takara). Capped polyadenylated Cas9 mRNA was produced by *in vitro* transcription (mMESSAGE mMACHINE T7 ULTRA kit®, Life Technologies) using as template the plasmid pMJ920 (Addgene 42234) linearized with BbsI and treated with Antarctic phosphatase (NEB). mRNA was purified using MEGAClear kit (Life Technologies). A solution of 300 ng/µl of mRNA and 100 ng/µl of each sgRNA was used for RNA microinjection. For ribonucleoprotein injection, Guide-it Recombinant Cas9 (Takara) and sgRNA/s were mixed to a final concentration of 300 ng/µl and 60 ng/µl, respectively, and incubated at 37 °C for 5 min to achieve ribonucleoprotein assembly prior to microinjection. Previous experiments were conducted to determine that the concentrations used did not reduce developmental rates compared to sham (buffer) injections. Microinjection was performed under a Nikon Diaphot TMD inverted microscope delivering 3–5 pl into the ooplasm using a filament needle.

### Embryo genotyping

CRISPR-injected embryos were kept in culture until day 9 after insemination. Zona pellucida was removed from unhatched blastocysts by incubation in acid PBS (pH 2) to avoid any residual spermatozoa and to facilitate subsequent enzymatic digestion. Zona-free blastocysts were individually placed at the bottom of a 0.2 ml PCR tube and stored at −80 °C until analysis. Each blastocyst was digested in 8 µl of Picopure® (ThermoFisher Scientific) for 1 h at 65 °C followed by inactivation at 95 °C for 10 min.

Genotyping of the intronic region was performed by clonal sequencing. PCR was performed on a 50 µl reaction containing 4 µl of the inactivated digestion product under the following conditions: 95 °C for 2 min; 39 × (95 °C for 20 s, 60 °C for 30 s, 72 °C for 40 s); 72 °C for 5 min; hold at 8 °C. PCR was performed using primers spanning the target sequence (Table S4). The PCR product from each blastocyst was purified using FavorPrep™ PCR Purification Kit (Favorgen). The purified product was Sanger sequenced and analyzed for the presence of indels. Consecutively, the purified PCR products from edited embryos, identified by a mixed sequence reaction around the target site (Fig. 3), were analyzed by clonal sequencing. For that aim, the purified PCR product was ligated into pMD20 T-vector (Takara) by Blunt/TA Ligase (NEB) and transformed into *Escherichia coli* DH5-α competent cells. For each embryo analysed, ten plasmids containing the insert were Sanger sequenced.

Genotyping of *PAEP* and *CSN2* target sequences was performed by deep sequencing. A first 30 cycle amplicon PCR was performed on 25 µl reaction containing 2 µl of the inactivated digestion product using the conditions detailed above and primers including Illumina overhangs (marked in italics in Table S4). Amplicons were purified by AMPPure XP beads (Beckman Coulter) and libraries were prepared by an index PCR which added Illumina adaptors and indexes identifying each embryo using Nextera XT (Illumina). Libraries were purified, pooled to 2 nM and sequenced on Illumina miSeq platform providing 250 bp paired-end sequencing reads. Individual alleles were identified following QC filtering, mapping to reference and variant calling.

Embryos showing more than two alleles by clonal or deep sequencing were considered mosaic. Statistical differences in genome edition and mosaicism rates were assessed by Chi-square test using SigmaStat software.

## Supplementary information

Supplementary information
