# Supplementary material for: Strategies to reduce genetic mosaicism following CRISPR-mediated genome edition in bovine embryos
Source: Sci Rep. 2019 Oct 17;9:14900. doi: 10.1038/s41598-019-51366-8 (PMC6797768; doi:10.1038/s41598-019-51366-8)
Supplement: Supplementary file 1 — Supplementary information [file 41598_2019_51366_MOESM1_ESM.pdf]

Strategies to reduce genetic mosaicism following CRISPR-mediated genome edition in bovine embryos

Lamas-Toranzo I[1], Galiano-Cogolludo B[1], Cornudella-Ardiaca F[1], Cobos-Figueroa J[1], Ousinde O[1], Bermejo-Álvarez P[1]*

[1]Animal Reproduction Department, INIA, Madrid, Spain.

*Corresponding author.

(Supplementary Information)

Table S1: Developmental rates following reduced gamete co-incubation times. Different letters indicate significant differences based on ANOVA ($p<0.05$).

IVF duration	*No. of oocytes inseminated*	*% Cleavage (n)*	*% Blastocysts (n)*
8 h	95	8.2 ± 0.7[a] (7)	0.0 ± 0.0[a] (0)
9 h	81	40.8 ± 2.7[b] (33)	12.4 ± 0.5[b] (10)
10 h	98	84.8 ± 2.8[c] (83)	29.6 ± 1.6[c] (29)
20 h	80	88.4 ± 4.6[c] (71)	31.1 ± 1.4[c] (25)

Table S2: Developmental rates following the alternative microinjection protocols tested. Different letters indicate significant differences based on ANOVA ($p<0.05$).

Group	No. of oocytes inseminated	% Cleavage (n)	% Blastocysts (n)
Non-injected 0 hpi	155	71.9 ± 0.7^{b} (111)	27.1 ± 2.1^{a} (40)
mRNA-injected 0 hpi	142	56.9 ± 3.8^{c} (69)	17.4 ± 3.3^{b} (25)
RNP-injected 0 hpi	146	57.1 ± 3.4^{c} (81)	15.1 ± 3.5^{b} (23)
Non-injected 10 hpi	48	84.9 ± 2.7^{a} (41)	27.8 ± 3.0^{a} (14)
mRNA-injected 10 hpi	155	$68.7 \pm 4.1^{b,c}$ (107)	17.6 ± 2.6^{b} (28)
Non-injected 20 hpi	160	92.9 ± 1.2^{a} (148)	34.4 ± 3.7^{a} (52)
mRNA-injected 20 hpi	140	$69.0 \pm 2.7^{b,c}$ (96)	$20.\ 0 \pm 1.7^{b}$ (28)

Table S3: Details for sgRNA used. Specificity and efficiency scores were provided by CRISPOR[46] (https://crispor.tefor.net)

gdRNA	Target sequence	Accesion number	CFD specificity score	Efficiency Fusi-score
Intron	GTCCTTACCGTTAATATTGT	NC_037338.1	79	36
PAEP	GAAGGGCCTGGATATCCAGA	NC_037338.1	80	65
CSN2	GAAGTGAAGGAGGAGCTGAA	NC_037333.1	54	63

Table S4: Genotyping primers used for clonal sequencing (intronic region) or deep sequencing analysis (*CSN2* and *PAEP*). Italic letters on PAEP and CSN2 primers indicate Illumina overhangs.

Primer	Sequence 5´-3´	Accesion number	WT product size
Intron F	CGAACCCTGCCACTACCATT	NC_037338.1	368
Intron R	CCCACCTCCCAACTGCTTAG	NC_037338.1	368
PAEP F	*TCGTCGGCAGCGTCAGATGTGTATAAGAGACAG*TCCTCCTGTATAAGGCCCCG	NC_037338.1	330
PAEP R	*GTCTCGTGGGCTCGGAGATGTGTATAAGAGACAG*GGGAATCCCAGACGTCACAG	NC_037338.1	330
CSN2 F	*TCGTCGGCAGCGTCAGATGTGTATAAGAGACAG*AGCCTGTTAACTAGTCTCGTGG	NC_037333.1	392
CSN2 R	*GTCTCGTGGGCTCGGAGATGTGTATAAGAGACAG*ACCACAAAATTAGCATGCCATTAAA	NC_037333.1	392

Figure S1: Examples of indels observed in edited embryos. Upper square shows wt sequence; target sequence is marked in red letters and PAM sequence in green letters. Lower squares show different alleles (indels in blue letters) present in three non-mosaic edited blastocysts.

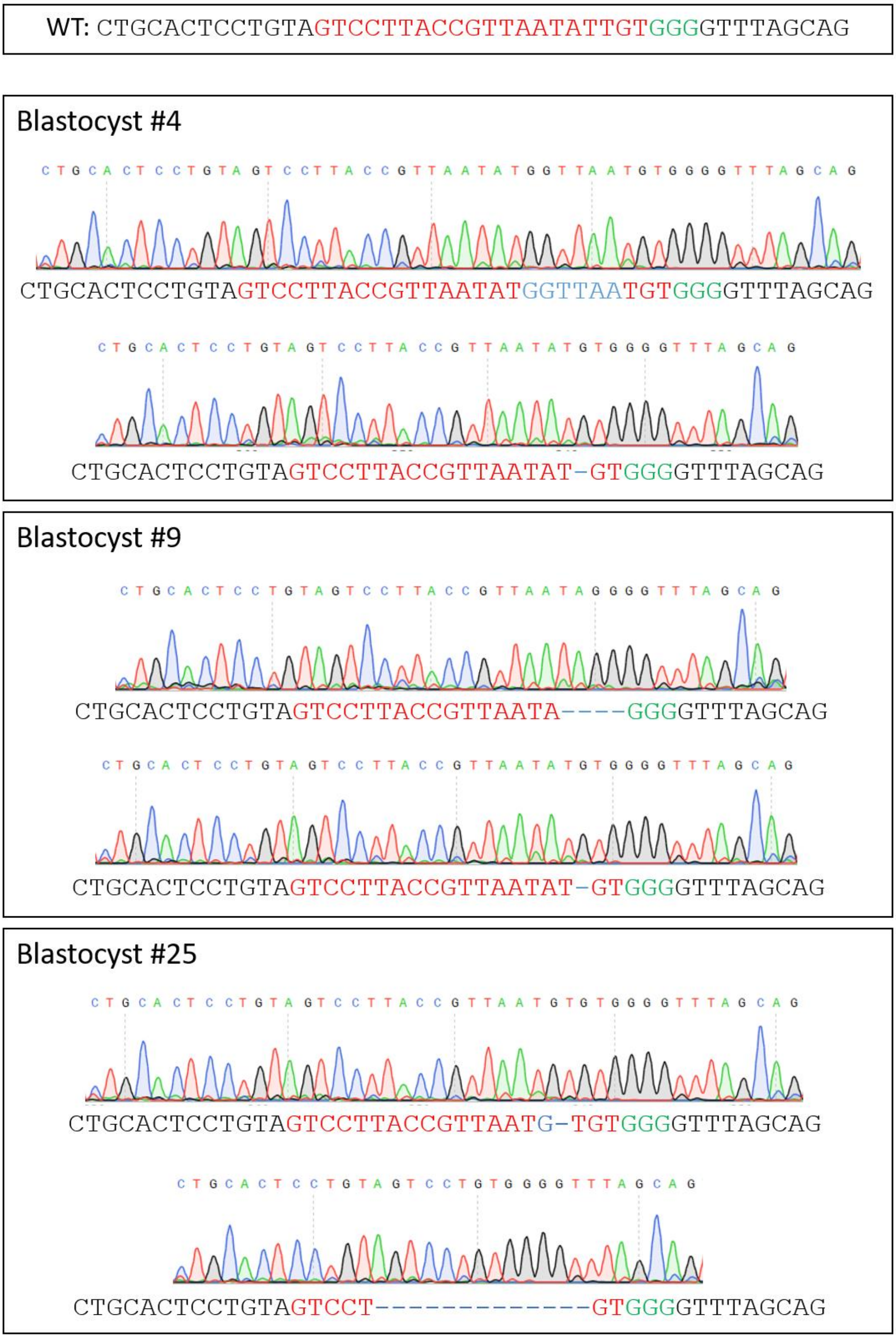

References

46 Haeussler, M. *et al.* Evaluation of off-target and on-target scoring algorithms and integration into the guide RNA selection tool CRISPOR. *Genome Biol* **17**, 148 (2016).
